# Deception about study purpose does not affect participant behavior

**DOI:** 10.1038/s41598-022-21972-0

**Published:** 2022-11-11

**Authors:** Zoe Rahwan, Barbara Fasolo, Oliver P. Hauser

**Affiliations:** 1 Max Planck Institute for Human Development, Berlin, Germany; 2 Department of Management, London School of Economics and Political Science, London, UK; 3 Department of Economics, University of Exeter, Exeter, UK

**Keywords:** Neuroscience, Psychology

## Abstract

The use of deception in research is divisive along disciplinary lines. Whereas psychologists argue that deception may be necessary to obtain unbiased measures, economists hold that deception can generate suspicion of researchers, invalidating measures and ‘poisoning’ the participant pool for others. However, experimental studies on the effects of deception, notably false-purpose deception—the most common form of experimental deception—are scarce. Challenges with participant attrition and avoiding confounds with a form of deception in which two related studies are presented as unrelated likely explain this scarcity. Here, we avoid these issues, testing within an experiment to what extent false-purpose deception affects honesty. We deploy two commonly used incentivized measures of honesty and unethical behavior: coin-flip and die-roll tasks. Across two pre-registered studies with over 2000 crowdsourced participants, we found that false-purpose deception did not affect honesty in either task, even when we deliberately provoked suspicion of deception. Past experience of deception also had no bearing on honesty. However, incentivized measures of norms indicated that many participants had reservations about researcher use of false-purpose deception in general—often considered the least concerning form of deception. Together, these findings suggest that while false-purpose deception is not fundamentally problematic in the context of measuring honesty, it should only be used as a method of last resort. Our results motivate further experimental research to study the causal effects of other forms of deception, and other potential spillovers.

## Introduction

Use of deception in the social sciences has long been controversial^1^. Deception is commonly defined as the intentional and explicit misleading of participants, as opposed to withholding information on hypotheses or experimental manipulations^2^. Revelations of the abuse of human subjects in deceptive experiments in both wartime (e.g., Nazi medical trials^3^) and peacetime (e.g., Milgram’s obedience experiments^4^) prompted major reforms in the ethical oversight of scientific research. The principles laid out in the American Psychological Association’s (APA) Code of Ethics^5^ and the Belmont Report^6^ continue to guide Internal Review Boards (IRBs) and ethics committees in the US and beyond^7^.

Deception practices differ along disciplinary lines^8–10^. Economists generally prohibit deception in experimental studies. Their objections are primarily motivated by practical concerns^11,12^, notably that deception will invoke mistrust and suspicion of the experimenter, both invalidating tests of economic theory and affecting participant behavior in future studies, with consequences including selective attrition^13–15^.

However, experimental evidence of these effects is scarce, with a few notable exceptions: Jamison et al.^14^ found that providing misleading information on partner identity (i.e., role deception) did not affect participants’ behavior in prisoners’ dilemma and dictator games in subsequent weeks, noting that only ~ 55% of participants returned after the initial phase of the study. They did, however, find some—albeit weak—indication of inconsistent behavior in a gambling task used to assess risk preferences, which may reflect reduced participant seriousness. A replication of this work which minimized the attrition rate between phases (8%) found no behavioral differences^12^. Another concern relates to selective attrition in future experiments. Jamison et al.^14^ found no differences between participants who had or had not been deceived, though they did find some weak evidence that deceived women were less likely to participate in future experiments than were undeceived women. Further, in a survey of predominantly American students, 25% reported that they would be less willing to participate in future experiments if they knew they had been deceived^16^.

Independent of the source of concern and whether it is supported by empirical evidence, there are tangible negative consequences for economics researchers using deception. Perhaps the most notable is that articles deploying deception are not eligible for publication in economics journals^17^—a policy that is sometimes explicitly stated (e.g., *Experimental Economics*^18^). In addition, more interdisciplinary journals, such as the *Proceedings of the National Academy of Sciences*, as well as grant funding bodies may reject research that contains or proposes deception^13,14,19,20^. Together, these may pose tangible barriers to career advancement^12^.

In psychology, in contrast, there is no blanket proscription of experimental deception. Rather, it has been argued that deception offers a means to study important yet uncomfortable aspects of the human condition (e.g., conformity, obedience)^21^ and that results may otherwise be biased by social desirability. Professional bodies such as the APA and many university IRBs discourage the use of deception, except where there is notable scientific value and non-deceptive alternatives are not viable^5,20^. However, the frequency of use of deception^1,22^ suggests that its use is not reserved to studies of particular importance. Further, there is evidence that, at least for studies on honesty and prosocial behavior, non-deceptive alternatives are commonly available but not used^23^.

Experimental deception comes in many forms and there is often disagreement as to what precisely can be identified as deception^16,24^. Sieber et al.’s^25^ taxonomy identifies eight types of deception, from stating a false purpose to not informing subjects that they are part of a study. We updated this taxonomy to reflect changes in experimental practices in recent decades, notably the proliferation of online experiments (Table S1). Here, we focus on the use of false-purpose deception—the most common form of deception in psychology^1^, where participants “may be given or be caused to hold false information about the main purpose of the study”^25^. An example would be stating that a study is about life and satisfaction when it actually aims to investigate how professional culture affects honesty^26^.

Variations in the stated experimental purpose may affect participant behavior in different ways. Participants who are informed that a study is on honesty or who suspect that this is the true experimental purpose may succumb to experimenter demand effects or social desirability bias, or simply behave more honestly amid an elevated sense of observation^27–30^. Alternatively, participants who suspect the experimenter of deceiving them about the study purpose may retaliate by increasing their dishonesty^27,31,32^. Another possibility is that there is simply no effect on behavior^33^.

Results from a meta-analysis of 565 experiments on individual (dis)honest behavior suggest that deceiving participants about the procedure leads to less dishonesty, though only in sender–receiver games (*k* = 165), which at a minimum involve role deception^30^. There was no evidence that use of deception affected honesty in coin-flip (*k* = 163) or die-roll (*k* = 129) tasks. For games involving collaborative dishonesty, a meta-analysis of 123 tasks, of which 16 entailed deception, found that the use of deception was associated with less dishonesty when all decision structures (e.g., joint, sequential) were pooled, but not when they were considered individually^34^. The authors speculated that participants who sense that they are being deceived may suspect that their behavior is not anonymous and that their actions will not in fact benefit the group, resulting in less collaborative dishonesty.

The present work was prompted by a methodological question that arose during the replication of a study^26,35^: While the original researchers had deployed false-purpose deception, we were only able to use incomplete disclosure due to concerns from field partners and the IRB. There appears to be a strong consensus that the use of false-purpose is a relatively benign form of deception—as distinct from other forms of deception^24^—and that it generates only low levels of concern among researchers, participants, and the general population, both in past decades^36^ and more recently^37,38^. Against this background, we expected minimal, if any, harm to arise to participants from our experiments.

To the best of our knowledge, Gallo et al.^39^ is the only previous study to experimentally manipulate deception solely in terms of false purpose. While the 3 (experimental disclosure) × 2 (task difficulty) design was pioneering at the time the study was conducted in the 1970s, it relied on a small (*n* = 120), unrepresentative sample (female, first-year psychology students at one university). The authors found no differences in outcomes due to experimental disclosure, although half of those deceived about the research purpose (conformity) reported being suspicious. Participants were not incentivized for accuracy.

Given the prevalence of false-purpose deception in academic research today, we add to this literature by providing the first new evidence in almost four decades: Our two studies adhere to today’s best research practices, as they are large-scale, highly powered, deploy incentivized measures, and are pre-registered. Our sample is drawn from a globally used research labor market—Amazon Mechanical Turk—that has come to play a major role in social and behavioral science since its launch in 2005^40^. The platform’s U.S. worker cohort is more representative than in-person samples, though less representative than panels^41^. Relative to the general population, U.S. Amazon Mechanical Turk workers tend to be younger, have European or Asian origin, be college educated, hold liberal political views, and hold agnostic or atheist views on religion^40^. The pay is commonly below the prevailing U.S. federal minimum wage^42^.

## Experiments

We conducted two pre-registered, highly powered experiments (*n* = 927, *n* = 1,209) on Amazon Mechanical Turk (MTurk) to examine how the type of disclosure regarding experimental purpose (true purpose, incomplete disclosure, or false purpose) affects behavior in two tasks commonly used to measure honesty: coin flipping (Study 1) and die rolling (Study 2). In Study 1, participants were asked to flip a coin 10 times, and each time they had an opportunity to win a reward of US5 cents, making a total maximum reward of US50 cents. Participants were informed of the winning outcome ahead of reporting their outcome, so were able to behave dishonestly. In Study 2, participants were asked to roll a die for which there were variable outcomes, ranging from US0 to USD50 cents^43^. We also added a fourth condition: absurd false purpose. Here, we told participants that we were studying “Juggling Clowns” in a deliberate effort to make them suspect deception. In Study 2, a number of measures were incentivized for accuracy: Participants were given financial rewards for accurately predicting peer behavior in the die-roll task and peer expectations with regard to the acceptability of false-purpose deception, as well as for accurate recall of the stated experimental purpose. Finally, in Study 2, we elicited non-incentivized views on deception in general, experience of deception on MTurk, and its experienced or anticipated spillovers (i.e., seriousness^37^, attention^39^, willingness to participate in future experiments^35,41^, suspicion of deception^33,44^, trust of researchers and science^45,46^, change in future behavior in similar and dissimilar tasks^47,48^).

## Results

**Figure 1:**
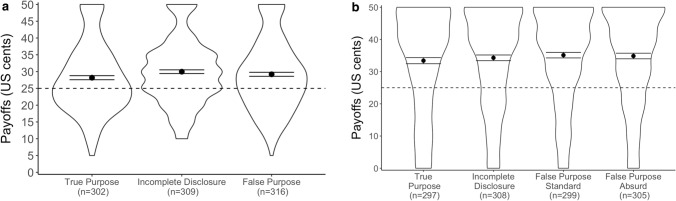
Actual payoffs and payoffs predicted by a fair coin (Panel **a**: Study 1) and die (Panel **b**: Study 2). Dishonesty occurred in every condition across both studies, as reflected in the skewness of distributions of reported payoffs and the averages deviating from that predicted by 10 rounds of coin flipping (Study 1) and a roll of a fair six-sided die: both USD25 cents. Differences between conditions were economically minimal and insignificant in most statistical analyses. Dots represent the average payoff for each condition. Error bars indicate standard errors of the mean. The dashed line represents the average outcome predicted by the theoretical distribution of a fair coin (Study 1) or six-sided die (Study 2).

Consistent with previous research^29,30^, comparison of our experimental results with the theoretical distributions of fair coins and dice (Figs. 1, S1) indicated dishonesty in the reporting of outcomes in both experiments and across all conditions (Wilcoxon and Kolmogorov–Smirnov tests, one-sided: all *P*s < 0.001). However, the level of cheating was limited, with participants forgoing an average of 42% and 31% of the maximum potential payoff (50 cents) in Study 1 and Study 2, respectively. In Study 2, we found, using incentivized measures, that participants expected their peers to cheat more than they actually did and to forgo only 13% of the maximum payoff (Fig. S2). Familiarity with the task was associated with a reduced likelihood (by 5 percentage points) of reporting a winning coin flip, but had no effect on reported die-roll outcomes (see Tables S2, S3).

Across both experiments, we found no robust evidence that the stated experimental purpose affected honesty (Tables S2, S3). Non-parametric pairwise tests did indicate a difference in honesty between the True Purpose and Incomplete Disclosure conditions in Study 1 *(W*_*two-sided*_ = 52,653, *P* = 0.005, *P*_Bonferroni-corrected_ = 0.05/3 = 0.017); however, the difference was economically negligible and no longer significant in probit models predicting the likelihood of reporting winning outcomes with and without control variables (Table S2).

Past self-reported experience of deception was not found to affect honesty in either study (Tables S2, S3), though it was associated with increased suspicion of deception (Tables S4, S5). Study 2 participants most often reported having experienced four different types of deception, while only 11% of participants reported no experience of deception (Fig. S3).

The level of suspicion of deception and attention to stated purpose did not explain the null findings either. In Study 1, general suspicion of deception rates was low (17%) and suspicion specifically regarding the stated experimental purpose was minimal (4%). Importantly, there were no differences in general suspicion rates between the conditions in Study 1, *H*(2) = 0.773, *P* = 0.680 (Table S4). Across conditions, however, suspicion of deception was associated with a 4 percentage point increase in the likelihood of reporting a winning outcome. In Study 2, suspicion of deception was higher in the Absurd False Purpose condition than in the other conditions (Table S5). Still, higher rates of suspicion were uncorrelated with the level of payoff (rho =   − 0.04, *P* = 0.203, Table S3) and there was no interaction between condition and level of suspicion in the regression predicting payoffs (Table S6).

In Study 2, we implemented incentivized measures of attention. We found that the majority of participants attended to the stated experimental purpose (*M*_*TotalSample*_ = 0.70, 95% CI = [0.68, 0.73]), and that those in the Absurd False Purpose condition were 16 percentage points (*P* < 0.001, 95% CI = [0.10, 0.22]) more likely to correctly identify the stated purpose of the experiment than those in the True Purpose condition (Table S7). However, attending to the stated purpose was not associated with any differences in honesty (Table S3).

**Figure 2:**
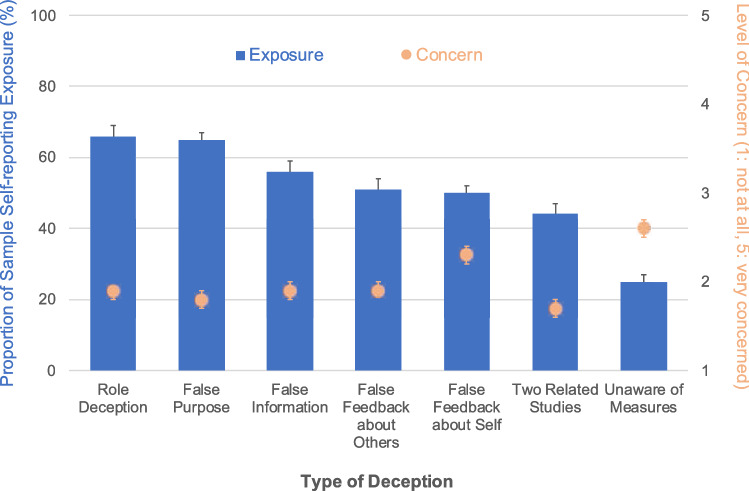
Participant past exposure to different types of deception and respective levels of concern (Study 2, *n* = 1,209). Role deception and false purpose are the most common types of deception participants report having been exposed to in prior experiments; being unaware of measures is the least common form. Level of concern is generally low across deception types, with the least concern being expressed for false-purpose deception and studies that present two related studies as being unrelated, and the highest concern for receiving false feedback about oneself and being unaware of measures in the experiment (e.g., filming, eye-tracking). Bars and spots reflect means; the error bars are 95% confidence intervals.

We also explored the possibility that the breach of trust from false-purpose deception was insufficient to evoke a response from participants. Survey data from Study 2 revealed low concerns about the general use of false-purpose deception—both in absolute terms and relative to other forms of deception (the only form of deception evoking lower concern, on average, was when two related studies were presented as unrelated). This is despite false-purpose deception (alongside role deception) being the most common form of deception experienced (Fig. 2). Further, when asked whether researchers should be allowed to use false-purpose deception on MTurk, 78% [95% CI = [0.75, 0.80]) of participants agreed. Together, these findings suggest that any breach of trust stemming from false-purpose deception may be insufficient to prompt a change in attitude or behavior. However, using an incentivized measure of social norms among MTurkers^49^, we found that only 54% (95% CI = [0.53, 0.56]) of participants thought that other MTurkers would agree with the use of false-purpose deception. Deception-naive participants, in particular, expected less tolerance among their peers, on average believing that peers would object to false-purpose deception (*M*_Deceived_ = 55.4, CI = [54.1, 56.8], *M*_Naive_ = 44.3, CI = [39.4, 49.2], *W*_two-sided_ = 1,430,754, *P* < 0.001).

Consistent with the relatively low levels of concern expressed regarding the use of deception, the majority of participants tended to expect relatively few adverse spillovers from exposure to deception, with the exception of suspicion (Fig. S4). In keeping with our experimental results indicating that increased suspicion will not always translate into changed behavior, our survey results also show that only a minority of participants reported or anticipated changing their behavior after exposure to deception (Table S8). Further, participants previously exposed to deception more commonly reported changed behavior for similar tasks than for different tasks, as has been speculated by some authors^50^. However, studies of spillovers from deception exposure on the same or similar tasks indicate a broad absence of any such effect^32^.

The majority of participants expected no change in future experimental behavior with regard to willingness to participate, attention, seriousness, and attitudes relating to trust in researchers and science in general. Nevertheless, notable minorities reported expecting or experiencing a loss of trust in researchers post-deception, especially among deception-naive participants *(n* = 132): 31%, CI = [24%, 39%], or paying increased attention in experiments post-deception. This is consistent with our experimental findings: Previously deceived participants were 13 percentage points more likely to correctly identify the experimental purpose (Table S6) and spent nearly double the time on the de-briefing page, measured in seconds (*M*_Deceived_ = 13.4, CI = [12.3, 14.5], *M*_Naive_ = 7.8, CI = [5.7, 9.9], *W*_one-sided_ = 1,447,670, *P* < 0.001). While previously deceived participants did have on average ~ 5 months more general experience on MTurk than naive participants (*M*_Deceived_ = 2.9, CI = [2.8, 3.1], *M*_Naive_ = 2.5, CI = [2.1, 2.8]*, W*_two-sided_ = 1,156,408, *P* < 0.001), it was exposure to deception rather than experience that was associated with differences in both attention to experimental purpose and suspicion (Tables S4, S6).

## Discussion

In two highly powered studies, we found no evidence that false-purpose deception affects participant honesty. Our results suggest that the findings of meta-analyses on studies using the die-roll and coin-flip tasks (e.g., 191 studies in^30^ and 82 studies in^29^) are unbiased by the form of stated experimental disclosure. This is broadly consistent with the absence of a correlational finding between the use of any sort of deception and honesty in Gerlach et al.^30^ for these tasks. The finding may be particularly helpful for researchers whose use of false-purpose deception is constrained, for example, by IRBs or field partners.

Even when we deliberately provoked suspicion regarding the experimental purpose (stating that the study was about “Juggling Clowns”), we found no discernable differences in honesty, consistent with the idea of a ‘good subject’^27^. Of course, suspicion of other forms of deception—which could be increased when an experiment is conducted incompetently—may have an effect on behavior. We found some—though not conclusive—evidence that the breach of trust caused by false-purpose deception is insufficiently severe to change participants’ behavior in future studies^20,36,51^. Likewise, we found no evidence that having been deceived in the past changed participants’ behavior in the honesty tasks, though it was associated with greater attention and suspicion. Nor did we find that our null finding could be explained by either inattention or variation in participant suspicion of experimenters engaging in deception.

We found evidence of generally low levels of concern about and broad tolerance for experimenter use of false-purpose deception. However, our measure of perceived peer acceptability of false-purpose deception, which was incentivized for accuracy, revealed that participants—especially those who were deception-naive—expected their peers to be much less tolerant. This indication of disquiet over the use of an arguably less egregious form of deception, together with the incomplete understanding of the spillovers of using experimental deception, leads us to concur with APA guidance that deception should be used as a method of last resort.

We hope that these studies, which revive work last conducted in the 1970s, but with a new type of experimental participant—the crowdsourced worker—inspire further investigation and interdisciplinary discussion of how different types of deception affect behavior in experiments and of the respective spillover effects. Thanks to our large sample sizes, the null effects reported are fairly precisely estimated, suggesting that these effects are robust. However, our results are limited to a single form of experimental deception (false purpose—one of the least egregious forms of deception) and behavior of interest (honesty), and to one crowdsourced labor platform (MTurk). We hope they will encourage reflection on how exposure to deception can be better documented, both on crowdsourced platforms and in laboratories, and that such documentation can spur further work seeking to improve research methods that can potentially affect the credibility of scientific findings.

## Materials and methods

These studies were pre-registered at AsPredicted.com before data collection began on 27 July 2017 and 9 March 2021, respectively. Ethical approvals were obtained from the Research Ethics Committee at the London School of Economics and Political Science (#000582 and #000921) and we confirm that all methods were performed in accordance with the relevant guidelines. As is standard practice, participants were asked to provide informed consent ahead of formally commencing the survey. Given the presence of deception, participants were debriefed regarding the nature and purpose of the deception.

Readers can access copies of the pre-registrations, survey files, data and R code at the Open Science Forum site: https://osf.io/f6gmb/?view_only=2ad7305cce094ff4a349850dcbcc304e. Further details on Methods can be found in Supplementary Information.

## Supplementary Information

Supplementary Information.

## Data Availability

The data sets generated by the current studies are available in the Open Science Forum repository, https://osf.io/f6gmb/?view_only=2ad7305cce094ff4a349850dcbcc304e.
