# Supplementary material for: Deception about study purpose does not affect participant behavior
Source: Sci Rep. 2022 Nov 11;12:19302. doi: 10.1038/s41598-022-21972-0 (PMC9652249; doi:10.1038/s41598-022-21972-0)
Supplement: Supplementary file 1 — Supplementary Information. [file 41598_2022_21972_MOESM1_ESM.docx]

**Supplementary Information for**

Deception about study purpose does not affect participant behavior

Zoe Rahwan^1,2^, Barbara Fasolo^2^, Oliver P. Hauser^3^

1: Max Planck Institute for Human Development, 2: London School of Economics and Political Science, 3: University of Exeter.

*Corresponding author: Zoe Rahwan

Email: zrahwan@mpib-berlin.mpg.de

**This PDF file includes:**

Supplementary information text – Methods and Materials

Figures S1 to S4

Tables S1 to S8

SI References

**Other supplementary materials for this manuscript include the following:**

Datasets S1 to S2

##

## Supplementary Information Text

###

## Methods and Materials

###

### Part 1: Study 1

####

#### A. Study Materials and Ethics approval

Our data, materials, and preregistration are available on the Open Science Framework (OSF):<https://osf.io/f6gmb/?view_only=2ad7305cce094ff4a349850dcbcc304e>. Approval for this research (including the use of deception) was provided by the London School of Economics Research Ethics Committee (reference #000582). Following the conclusion of the study, all participants were fully de-briefed via a message sent separately on the MTurk messaging service.

#### B. Sample and Recruitment

This study was run on 27 July 2017 on Amazon Mechanical Turk using U.S. participants. We strived to recruit 900 participants across three conditions (i.e., 300 participants per condition). The sample size was determined from previous studies conducted using the same 10-round coin-flipping task, to enable the detection of a 0.5 difference in total reported coin-flip wins between two conditions, the minimum difference to result in a change in payoff. In the end, a total of 927 participants completed the survey (mean age = 36 years, age range = 18–76, 423 men, 498 women, 4 identifying as ‘other’). Note that we deliberately did not seek a nationally representative sample as our population of interest was that of MTurk.

#### C. Motivation and Procedure

This initial study was motivated by differences in stated experimental purpose in undertaking a replication ^1^ of a highly cited field study ^2,3^. The original study used a false purpose (“Life and Satisfaction)”, while the replication studies used incomplete disclosure (“Norms and Attitudes”) due to differing IRB standards and field partner constraints. Because the replication studies did not find the same results as the original study, one hypothesis was that this may have been due to differences between studies in the disclosed experimental purpose. However, as the initial and replication studies were conducted at different times and in different populations, it was not possible to isolate the role of the disclosed experimental purpose.

The present study therefore studied this question systematically by varying the stated purpose across three randomized conditions. When participants joined the study, they were presented with a welcome page that randomly presented one of three types of experimental purpose: true (“Honesty”), incomplete disclosure (“Norms and Attitudes”), and false (“Life and Satisfaction”). After consenting to join the study, all participants were asked questions about happiness, satisfaction, and leisure activities. We included these questions for all conditions so that even participants in the ‘false’ condition (“Life and Satisfaction”) could plausibly believe that we, the researchers, were interested in these outcomes; our main interest was, of course, their behavior in the honesty task described below. (This design feature is also consistent with the original study we were attempting to replicate.)

Next, the key outcome variable—honesty—was measured in a 10-round “coin-flipping” task. The multi-round coin-flipping task works as follows ^2^: In each round, participants could win a US5 cent bonus for reporting a winning coin flip. Participants were then asked to submit their own answer when asked what the coin flip revealed. This self-report meant that there was an opportunity to cheat: Participants were informed of the winning outcome ahead of submitting their answers, giving them an opportunity to cheat to receive up to a maximum payoff of US 50 cents if they cheated in every one of the 10 rounds. Given the binomial distribution of a fair coin toss, we can detect both the presence of cheating and the difference in cheating across conditions.

We then took an implicit measure of moral feelings after the coin-flip task by asking participants to solve word fragment puzzles. Four of the six puzzles could be solved with ‘moral’ words (pure, virtue, moral, and ethical), inspired by ^4^. We were interested to see if being able to spot the moral words varied across the experimental conditions. Participants also completed additional measures (as per Cohn et al., 2014) to capture self-reported materialism, altruism, competitiveness, other-regarding concerns in general and with regard to their work.

Basic demographic information was collected (age, gender, education, region of US residence, MTurk experience). At the end of the survey, we applied a ‘prompted’ measure of suspicion ^5^ to determine whether individuals were suspicious about being deceived in this study and about what they thought they were deceived about. It has long been believed (e.g. Kelman, 1967 ^6^)that the use of deception can increase the suspicion of participants towards researchers. That said, Krasnow et al. ^5^ did not find that suspicion was linked to past exposure to deception. We took measures of past perceived deception on MTurk and its nature, experience in coin-flipping tasks, recency of the last coin-flipping task, and participants’ self-reported emotions after and the speculated purpose of completing such tasks.

#### D. Analytical Approach

We conducted analysis using non-parametric measures, due to the skewed nature of our main measure of interest, i.e., the self-reported number of winning coin tosses. We also conducted linear regression analyses, modelling both total winning coin tosses and the reporting of a winning coin toss, clustering standard errors at the level of participants across rounds and controlling for a range of demographic and other variables. This is as per the pre-registration.

### Part 2: Study 2

#### A. Study Materials and Ethics approval

Our data, materials, and preregistration are available on the OSF:<https://osf.io/f6gmb/?view_only=2ad7305cce094ff4a349850dcbcc304e>. Approval for the use of deception was provided by the London School of Economics Research Ethics Committee (reference #000921). Following the conclusion of the study, all participants were fully de-briefed within the survey.

#### B. Sample and Recruitment

Study 2 was launched on March 9 2021 on Amazon Mechanical Turk using U.S. participants. We strived to recruit 1,200 participants (i.e., 300 per condition in line with the first experiment). This also ensured comparable statistical power to experiments using the same die-roll task ^7,8^. In the end, 1,209 participants completed the survey (mean age = 40 years, age range = 18–79, 556 men, 636 women, 10 non-binary individuals, and 7 preferring not to answer). Note that we deliberately did not seek a nationally representative sample as our population of interest was that of MTurk.

#### C. Procedure

We extended Study 1 to explore whether (i) deliberately provoking suspicion via stated experimental purpose (the fourth condition claimed to be about “Juggling Clowns”) can affect honesty behavior and (ii) whether inattention or lack of seriousness in completing the survey could explain the insensitivity to stated experimental purpose.

Similar to Study 1, we recruited participants from MTurk, posting that the study was about “judgement and decision making.” When participants joined the study, they were presented with a welcome page that randomly presented one of four types of experimental purpose: true, incomplete disclosure, and two false conditions (either a standard false purpose: “Life and Satisfaction”; or an absurd purpose: “Juggling Clowns”). On the second page, participants were presented with a standard consent form that reiterated the stated experimental purpose.

In keeping with Study 1, participants first completed questions about life and satisfaction, followed by the honesty task. In Study 2, however, we chose a slightly different measure of honesty to ensure that our earlier results were not simply an artifact of the honesty measure used. In Study 2, we therefore used a one-shot die roll with variable payoffs ^7^. Participants were asked to use a real (physical) die or visit a die-rolling site, and roll the die to ensure that it is fair ^9^. Once satisfied, participants were asked to roll the die and self-report the die-roll outcome. Participants were aware of the variable bonus depending on their self-report: US10 cents for reporting 1, US20 cents for 2, ... US50 cents for 5, and US0 cents for 6. Given the equal probability of each outcome, at a group level, comparison can be made to a uniform distribution to assess the presence and degree of dishonesty, but dishonesty cannot be identified at an individual level.

Next, we assessed the presence of suspicion again using ‘prompted’ measures from Krasnow et al. ^5^. Ahead of this, we provided reassurances that answers would not affect compensation, as per Blackhart el al. ^10^. Participants were asked if they believed “they were intentionally misled about any part of this study” (yes/no), and how confident they were in their belief (7-point scale, with 1 = “I am positive I was not deceived” and 7 = “I am positive I was deceived”). We then asked what they believed that had been deceived about (free text response).

Participants were then asked to recall the stated purpose of the study as our manipulation check. They were offered a US10 cent bonus for selecting the correct response from a list which noted the four conditions and “I don’t know.” For participants who did not choose “I don’t know,” we asked them what they thought the true purpose of the study was, offering the following options: the same as the stated purpose, “I don’t know”, or “Other” (free text response). Of the 282 participants choosing “Other”, 247 (88%) correctly reported a belief that we were assessing honesty.

Next, we assessed individual judgements and peer expectations with regards to researcher use of false purpose on MTurk. We asked whether individuals thought it should be permissible for researchers to use deception in studies (yes/no) and how many (out of 100 MTurker peers) they thought would believe it to be permissible (participants chose from 11 increments from 0 to 100). A reward of US10 cents was offered for the correct answer (rounded to the nearest 10) to further mitigate social desirability effects ^11^.

Participants were then asked to complete four questions regarding trust in researchers and science in general. Specifically, we asked how much participants trusted researchers they had previously worked for on MTurk, science in general, and that we and other researchers would pay promised bonuses.

Next, we introduced a section regarding experience, concerns, and expected spillovers from deception in general. We adapted the definitions of deception from Sieber et al. ^12^ and updated them to reflect a major change in social science research—the proliferation of online experiments enabled by crowdsourced platforms ^13^ (see Table 1). We then asked if participants recalled being debriefed regarding a deception (yes/no/unsure). For those answering “yes” or “unsure,” we probed which type of deception they had been exposed to.

All participants were asked about their level of concern—philosophical or practical—about the seven different types of deception (5-point scale; “not at all concerned”, “moderately concerned”, “very concerned”). Based on previous exposure to deception, we probed what spillovers they had either experienced or would anticipate experiencing; behavior in similar tasks in future studies (change/no change), behavior in different tasks in future studies (change/no change), level of trust in researchers and science, willingness to participate in future studies, level of attention, seriousness, and suspicion in future studies (decreased, no change, increased). Open text questions on other effects of deception and thoughts about researchers’ use of deception were then posed.

We asked about die-roll experience (0–100, more than 100 tasks), what participants thought the purpose of the die-rolling task was (open text). After providing a reassurance that it would not affect any payments ^10^, we asked how serious participants were in undertaking this survey (5-point scale, anchored with “not at all” and “very serious.”)

Finally, we collected demographic information (age, gender, education, relative income, income, political and religious preferences ^14^), and data related to MTurk experience (number of years, number of HITs, share of academic study HITs, importance of their work on MTurk for income and for generating a sense of purpose (5-point scale, anchored with “not at all important” and “very important”).

#### D. Analytical Approach

We conducted analyses using the approaches outlined in Study 1. That is, we used non-parametric tests, due to the skewed nature of our main measure of interest, i.e., the die-roll outcome. The die-roll outcome is presented in terms of payoffs for ease of comprehension. We also conducted linear regression analyses, modelling the payoff against treatment, controlling for a range of demographic and other variables. This is as per the pre-registration.

## Figures

a.
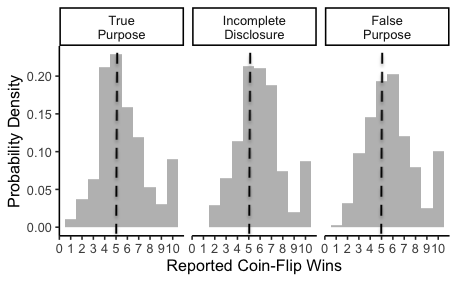
b.
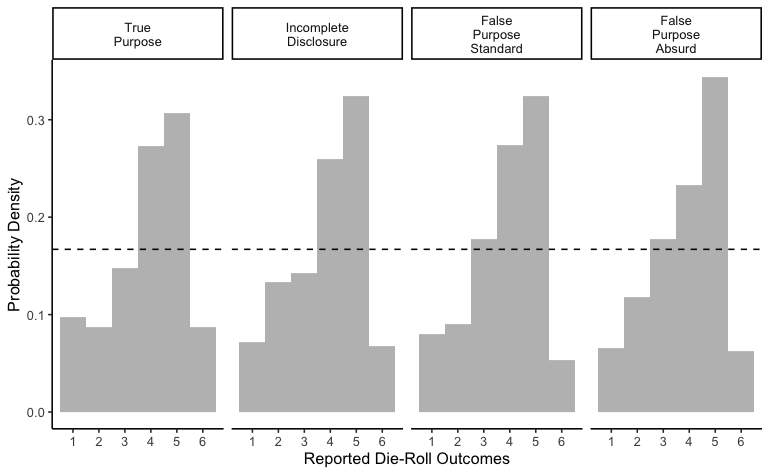


### Figure S1. Distributions of reported coin-flip wins (Study 1, *Left*) and die-roll outcomes (Study 2, *Right*). a. Probability densities of the reported outcomes from the coin-flip task (0–10) by condition. Cheating was detected relative to the theoretical distribution, the mean of which is marked with a dotted line. b. Probability densities of the reported outcomes from the die-roll task (1–6) by condition. The dashed line represents what would be expected in the absence of dishonesty: a uniform distribution with each die-roll outcome having a probability of ⅙.


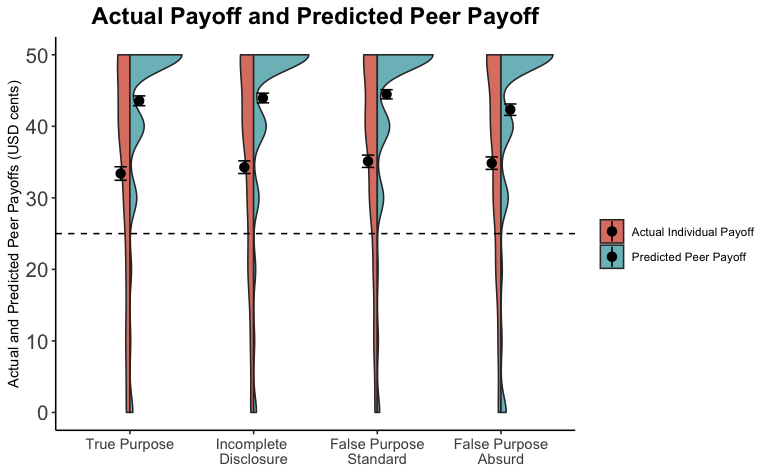


**Figure S2. Actual payoffs earned by individual participants and predictions of peer payoffs in the die-roll task (Study 2).** The average payoffs earned by individuals in each condition were above the average of the theoretical distribution of a fair six-sided die—shown by the dashed line—and reflect dishonesty in participants’ reporting of outcomes. In each condition, participants predicted that their peers would behave more dishonestly than was actually observed. Dots represent the average payoff for each condition. Error bars indicate standard errors of the mean. The violin plots present the distribution of payoffs for each condition.

###
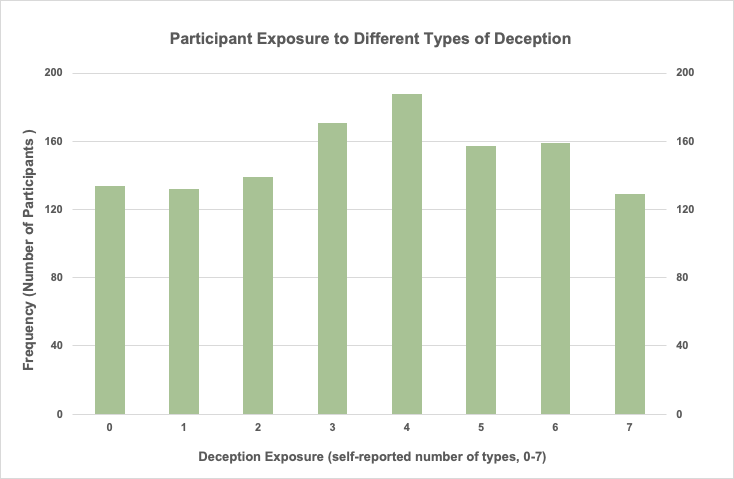


### Figure S3. The number of types of deception experienced by individual participants on MTurk (Study 2). We surveyed participants on seven types of deception. Only 134 participants from a sample of 1,209 (11%) reported having experienced no deception on MTurk; 129 participants (11%) reported having experienced all seven types of deception on MTurk.

##

###
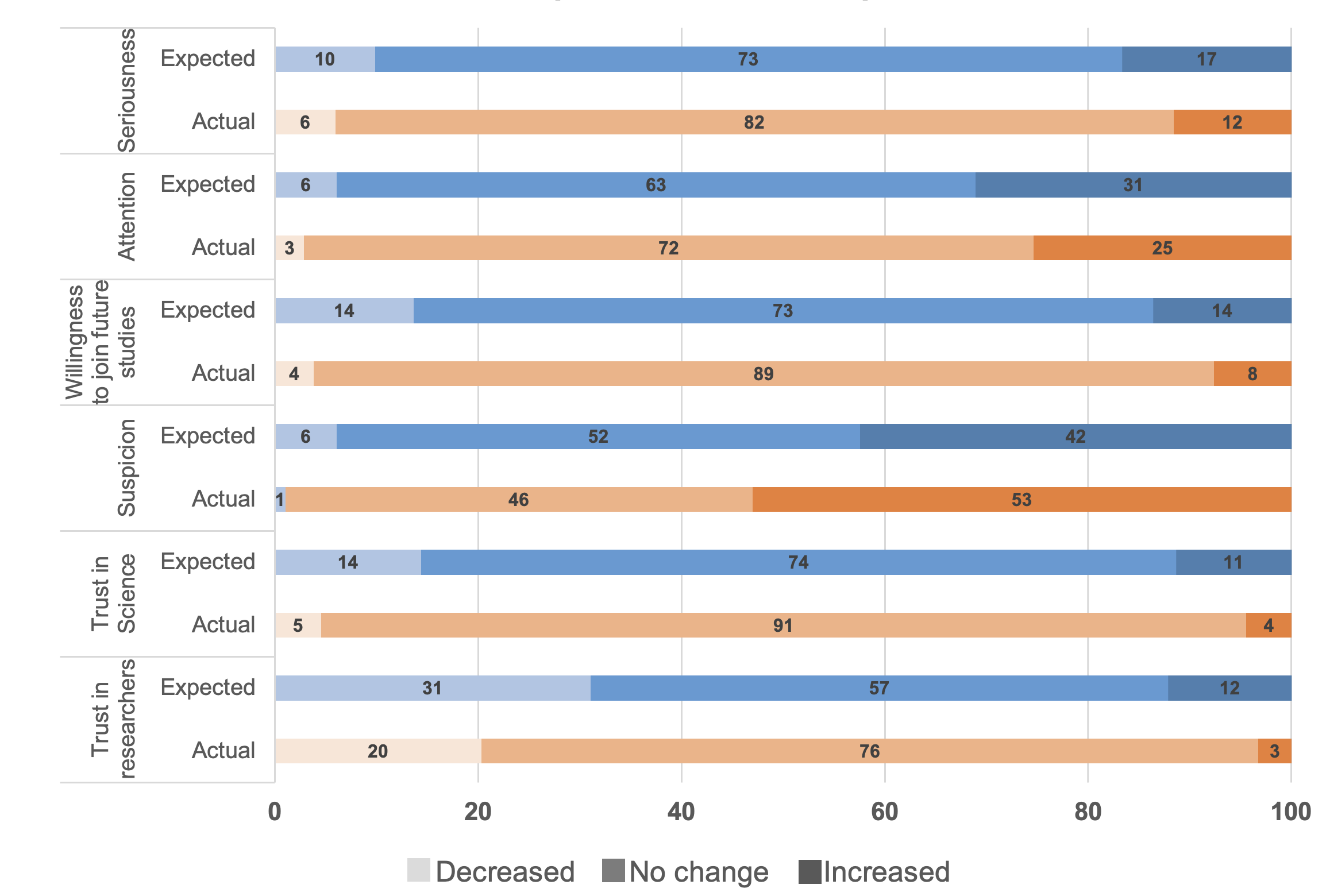


### Figure S4. Survey results on actual spillovers experienced by those previously exposed to deception (*n* = 1,077) and spillovers expected by those who did not recall being exposed to deception (*n* = 132) (Study 2). The majority of participants did not experience or expect spillovers from deception. The one exception relates to suspicion, where those previously exposed to deception, but not naive participants, on average reported increased suspicion. The figure shows the proportion of responses (decreased, no change, increased) for each type of spillover.

###

## Tables

### Table S1. Revised Definitions of Deception

| **Original Definitions (Sieber et al, 1995) as described by Hertwig & Ortman (2008)** | **Revised Definitions, adapted to be meaningful from the perspective of an experimental participant** |
| --- | --- |
| **False purpose.** Participants are given, or cause to hold, false information about the main purpose of the study. | **False purpose:** are given, or cause to hold, false information about the main purpose of the study. This is distinct from being given incomplete information regarding the purpose of the study. |
| **Bogus device.*** Participants are given false information concerning stimulus material. | **False information in stimulus materials.** Participants are given information that is untrue in an experimental task. For example, participants are given false answers of a potential dating partner, participants are incorrectly informed that a human rather than an Artificial Intelligence (AI) generated text/image/sound/video, inaccurate information regarding rewards is provided.** |
| **Role deception**. Participants interact with participants about whose identity they have been given false information. | **Role deception.** Participants interact with others in an experimental setting about whose identity they have been given false information. For example, confederates, false claims regarding being paired with a partner, including whether the partner is artificially intelligent. |
| **False feedback regarding self.** Participants are given false feedback about themselves. | **False feedback regarding self.** Receiving false feedback about yourself. |
| **False feedback regarding others.** Participants are given false feedback about another person. | **False feedback regarding others.** Receiving false feedback about others. |
| **Two related studies.** Two related studies are presented as unrelated | **Two related studies.** Two related studies or tasks are presented as unrelated |
| **Unaware of measure.** Participants are kept unaware that a study is in progress at the time of manipulation or measurement, or unaware of being measured (e.g., videotaped) | **Unaware of measure.** Participants are kept unaware that a study is in progress at the time of manipulation or measurement, or unaware of being measured (e.g., filming, eye-tracking). This excludes measures such as hidden click counts and question timers in online surveys. |
| **Unaware of participation.** Participants are kept unaware of being subjects in research. | (see above) |

*Bogus physical devices (e.g., lie-detector machines, Crutchfield apparatus) are of lesser concern in current experimental settings, particularly online. **Qualitative review of our survey results (specifically, the Study 1 open question about what people suspected they may have been deceived about in our study, and the Study 2 question about the nature of past deception) suggests that misleading information regarding rewards paid in a task is another relevant example, including rewards paid to charity on the participant’s behalf.

### Table S2. Predicting Winning Coin-Flips: Marginal Effects from Probit Models in Study 1

| **Dependent Variable:**  Likelihood of Reporting a Winning Coin Toss | **Predictors** | **Model (1)** | **Model (2)**  - with experimental controls | **Model (3)**  - with demographics |
| --- | --- | --- | --- | --- |
| **Treatment**  (False Purpose omitted) | Incomplete Disclosure | 0.02  [-0.02, 0.05] | 0.02  [-0.01, 0.05] | 0.02  [-0.01, 0.05] |
|  | True Purpose | -0.02  [-0.05, 0.01] | -0.02  [-0.05, 0.01] | -0.02  [-0.05, 0.01] |
|  | MTurk experience (years) |  | 0.01*  [ 0.00, 0.02] | 0.01*  [ 0.00, 0.02] |
| **Suspicion of Deception** | Current Experiment (dummy) |  | 0.04*  [0.00, 0.08] | 0.04*  [0.00, 0.08] |
|  | Past Experiment (dummy) |  | -0.02  [-0.05, 0.01] | -0.01  [-0.04, 0.02] |
|  | Coinflip task experience (# tasks) |  | 0.00  [-0.00, 0.00] | 0.00  [-0.00, 0.00] |
|  | Coinflip purpose known |  | -0.05***  [-0.08, -0.02] | -0.05***  [-0.08, -0.02] |
|  | Age (years) |  |  | -0.00*  [-0.00, -0.00] |
|  | Male |  |  | 0.03*  [ 0.01, 0.06] |
|  | Higher Education |  |  | -0.02  [-0.05, 0.01] |
| **Region** | North East |  |  | -0.00  [-0.04, 0.04] |
| (Mid-West omitted) | Pacific |  |  | 0.12  [-0.02, 0.26] |
|  | South |  |  | -0.04*  [-0.08, -0.01] |
|  | West |  |  | -0.02  [-0.06, 0.02] |
| **AIC** |  | 12,596 | 12,563 | 12,489 |
| **Log Likelihood** |  | -6,295 | -6,273 | -6,230 |
| **Deviance** |  | 12,590 | 12,548 | 12,459 |
| **Num. obs.** |  | 9,270 | 9,270 | 9,250 |

Note: Responses for individuals are clustered and standard errors are robust. Figures in brackets denote 95% confidence intervals of the estimate. * p<0.05 ** p<0.01 *** p<0.001

### Table S3: Predicting Die-Roll Payoffs: Coefficients from Linear Regression Models in Study 2

| **Dependent Variable:** Payoff from die-roll outcome (USD) | **Predictors** | **Model (1)** | **Model (2)**  - with experimental controls | **Model (3)**  - with demographics |
| --- | --- | --- | --- | --- |
|  | Intercept | 33.40^***^  [31.64, 35.16] | 36.44^***^  [32.96, 39.91] | 39.91^***^  [34.70, 45.12] |
| **Treatment**  (True Purpose omitted) | Incomplete Disclosure | 0.89  [-1.58, 3.35] | 0.68  [-1.81, 3.18] | 0.61  [-1.87, 3.09] |
|  | False Purpose – Absurd | 1.45  [-1.02, 3.93] | 1.65  [-0.87, 4.17] | 1.47  [-1.04, 3.98] |
|  | False Purpose – Standard | 1.72  [-0.77 4.20] | 1.62  [-0.89 4.12] | 1.62  [-0.88 4.11] |
|  | MTurk experience (years) |  | -0.19  [-0.59 0.21] | -0.07  [-0.48 0.33] |
|  | Die-roll experience (# tasks) |  | 0.09^**^  [0.02, 0.15] | 0.08^*^  [0.01, 0.14] |
|  | Suspicion of deception (dummy) |  | -1.77  [-4.13, 0.58] | -2.04  [-4.38, 0.30] |
|  | Past experiment deception (dummy) |  | -1.32  [-3.55, 0.90] | -0.72  [-2.97, 1.52] |
|  | Stated purpose identified (dummy) |  | -0.76  [-2.73, 1.22] | -0.40  [-2.37, 1.57] |
|  | Die-roll purpose identified (dummy) |  | -0.89  [-2.67, 0.89] | -1.04  [-2.82, 0.73] |
|  | Age (years) |  |  | -0.12^***^  [-0.19, -0.05] |
|  | Male |  |  | 1.34  [-0.46, 3.13] |
|  | Higher Education |  |  | 0.72  [-1.24, 2.67] |
|  | Relative Income |  |  | 0.16  [-0.24, 0.56] |
|  | Political Preference (economic) |  |  | 0.02  [-0.24, 0.27] |
|  | Religiosity |  |  | -0.39  [-0.82, 0.03] |
| **Observations** |  | 1,209 | 1,191 | 1,190 |
| **R^2^** |  | 0.002 | 0.014 | 0.032 |

Figures in brackets denote 95% confidence intervals of the estimate. * p<0.05 ** p<0.01 *** p<0.001

### Table S4: Predicting Suspicion: Marginal Effects from Probit Model (Study 1)

| **Dependent Variable:** Suspicion of Deception in Current Experiment |  |
| --- | --- |
| *Predictors* | *Estimates* |
| Incomplete Disclosure | -0.03  [-0.08, 0.03] |
| True Purpose | 0.00  [-0.06, 0.05] |
| Experience in Coin-Flip tasks (#) | 0.00  [-0.00, 0.01] |
| Deceived in past (dummy) | 0.17***  [0.12, 0.21] |
| MTurk experience (years) | -0.01  [-0.03, 0.00] |
| **AIC** | 805.40 |
| **Log Likelihood** | -396.70 |
| **Deviance** | 793.40 |
| **Number of Observations** | 927 |
| Note: False Purpose variable omitted. Figures in brackets denote 95% confidence intervals of the estimate  ** p<0.05 ** p<0.01 *** p<0.001* | |
|  | |

### Table S5: Predicting Suspicion: Marginal Effects from Probit Model (Study 2)

| **Dependent Variable:** Suspicion of Deception in Current Experiment |  |
| --- | --- |
| *Predictors* | *Estimates* |
| True Purpose | 0.05  [-0.02, 0.11] |
| Incomplete Disclosure | -0.03  [-0.09, 0.03] |
| Absurd False Purpose | 0.09**  [0.03, 0.16] |
| Experience with Die-Rolling tasks | -0.00  [-0.00, 0.00] |
| Deceived in past (dummy) | 0.10***  [0.04, 0.15] |
| MTurk experience (years) | -0.02**  [-0.03, -0.01] |
| **AIC** | **1077.9** |
| **Log Likelihood** | **-531.96** |
| **Deviance** | **1063.9** |
| **Number of Observations** | **1191** |
| Note: (Standard)False Purpose variable omitted. Figures in brackets denote 95% confidence intervals of the estimate  ** p<0.05 ** p<0.01 *** p<0.001* | |

### Table S6: Predicting Die-Roll Payoffs: Coefficients from Linear Regression Models with suspicion interactions in Study 2

| **Dependent Variable: Payoff (USD)** | **Model (1)** | **Model (2)** |
| --- | --- | --- |
| *Predictors* | *Estimates* | *Estimates* |
| Intercept | 33.68^***^  [31.73, 35.63] | 32.91^***^  [30.86, 34.96] |
| Incomplete Disclosure | 1.08  [-1.60, 3.77] | 1.13  [-1.57, 3.84] |
| False Purpose – Absurd | 1.26  [-1.54, 4.05] | 1.10  [-1.71, 3.90] |
| False Purpose – Standard | 1.99  [-0.74, 4.71] | 1.86  [-0.88, 4.60] |
| Suspicion of Deception | -1.50  [-6.03, -3.04] | -1.00  [-5.68, -3.68] |
| Incomplete Disclosure X  Suspicion of Deception | -2.45  [-9.44, -4.54] | -2.57  [-9.56, -4.43] |
| False Purpose – Absurd X  Suspicion of Deception | 1.16  [-4.93, 7.24] | 1.66  [-4.43, 7.76] |
| False Purpose – Standard X  Suspicion of Deception | -2.31  [-9.06, 4.45] | -2.13  [-8.88, -4.62] |
| Die-Roll Experience (# tasks) |  | 0.09^**^  [0.02, 0.16] |
| Suspicion of Deception * Die-Roll Experience (# tasks) |  | -0.05  [-0.22, 0.12] |
| Observations | 1209 | 1191 |
| R^2^ | 0.006 | 0.011 |

Note: True Purpose variable omitted. Figures in brackets denote 95% confidence intervals of the estimate

** p<0.05 ** p<0.01 *** p<0.001*

### Table S7: Manipulation Check: Marginal Effects from Probit Models in Study 2

| **Dependent Variable:**  Correctly Identifying  Stated Purpose | **Model (1)** | **Model (2)** | **Model (3)** |
| --- | --- | --- | --- |
| *Predictors* | *Estimates* | *Estimates* | *Estimates* |
| Incomplete Disclosure | -0.08*  [-0.15, -0.01] | -0.08*  [-0.15, -0.01] | -0.08*  [-0.15, -0.01] |
| False Purpose – Absurd | 0.16***  [ 0.10, 0.22] | 0.16***  [0.10, 0.22] | 0.16***  [0.10, 0.23] |
| False Purpose – Standard | 0.03  [-0.04, 0.10] | 0.03  [-0.04, 0.10] | 0.03  [-0.04, 0.10] |
| Past Deception (dummy) | 0.13**  [0.04, 0.22] | 0.14**  [0.05, 0.23] | 0.11  [-0.02, 0.25] |
| MTurk experience (years) |  | -0.01*  [-0.02, -0.00] | -0.02  [-0.06, 0.02] |
| Past Deception X MTurk experience |  |  | 0.01  [-0.03, 0.05] |
|  |  |  |  |
| **AIC** | 1425.6 | 1422.0 | 1424.0 |
| **Log Likelihood** | -707.8 | -705.1 | -705.0 |
| **Deviance** | 1415.6 | 1404.2 | 1410.0 |
| **Number of Observations** | 1209 | 1209 | 1209 |
| Note: True Purpose variable omitted. Figures in brackets denote 95% confidence intervals of the estimate  ** p<0.05 ** p<0.01 *** p<0.001.* | | | |

### Table S8. Experienced and Anticipated Spillovers from Deception on Task Behavior in Study 2

| **Proportion of Total (%)** | Actual effects reported by those having experienced deception  (*n* = 1077) | | Anticipated effects reported by those having no prior exposure to deception  (*n* = 132) | |
| --- | --- | --- | --- | --- |
|  | ***No change*** | ***Change*** | ***No Change*** | ***Change*** |
| Similar tasks | 74  [71, 77] | 26  [23, 29] | 71  [63, 79] | 29  [21, 37] |
| Different tasks | 81  [78, 83] | 19  [17, 22] | 69  [61, 77] | 31  [23, 39] |

Note: 95% confidence intervals are presented in brackets.

##

## SI References

1. Rahwan, Z., Yoeli, E. & Fasolo, B. Heterogeneity in banker culture and its influence on dishonesty. *Nature* **575**, (2019).

2. Cohn, A., Fehr, E. & Marechal, M. A. Business culture and dishonesty in the banking industry. *Nature* **516**, 86–89 (2014).

3. Altmetric – Business culture and dishonesty in the banking industry. https://www.altmetric.com/details/2905465 (2020).

4. Gino, F., Schweitzer, M. E., Mead, N. L. & Ariely, D. Unable to resist temptation: How self-control depletion promotes unethical behavior. *Organ. Behav. Hum. Decis. Process.* **115**, 191–203 (2011).

5. Krasnow, M. M., Howard, R. M. & Eisenbruch, A. B. The importance of being honest? Evidence that deception may not pollute social science subject pools after all. *Behav. Res. Methods* **52**, 1175–1188 (2020).

6. Kelman, H. C. Human use of human subjects: the problem of deception in social psychological experiments. *Psychol. Bull.* **67**, 1–11 (1967).

7. Fischbacher, U. & Föllmi-Heusi, F. Lies in disguise—an experimental study on cheating. *Journal of the European Economic Association* **11**, 525–547 (2013).

8. Kajackaite, A. & Gneezy, U. Incentives and cheating. *Games Econ. Behav.* **102**, 433–444 (2017).

9. Shalvi, S., Dana, J., Handgraaf, M. J. J. & De Dreu, C. K. W. Justified ethicality: Observing desired counterfactuals modifies ethical perceptions and behavior. *Organ. Behav. Hum. Decis. Process.* **115**, 181–190 (2011).

10. Blackhart, G. C., Brown, K. E., Clark, T., Pierce, D. L. & Shell, K. Assessing the adequacy of postexperimental inquiries in deception research and the factors that promote participant honesty. *Behav. Res. Methods* **44**, 24–40 (2012).

11. Bicchieri, C. *Norms in the Wild: How to Diagnose, Measure, and Change Social Norms*. (Oxford University Press, 2016).

12. Sieber, J. E., Iannuzzo, R. & Rodriguez, B. Deception Methods in Psychology: Have They Changed in 23 Years? *Ethics Behav.* **5**, 67–85 (1995).

13. Chandler, J., Rosenzweig, C., Moss, A. J., Robinson, J. & Litman, L. Online panels in social science research: Expanding sampling methods beyond Mechanical Turk. *Behav. Res. Methods* **51**, 2022–2038 (2019).

14. Huang, K., Bernhard, R. M., Barak-Corren, N., Bazerman, M. H. & Greene, J. D. Veil-of-ignorance reasoning mitigates self-serving bias in resource allocation during the COVID-19 crisis. *Judgm. Decis. Mak.* **16**, (2021).

## Datasets

**Dataset S1 (separate file).**  Study 1 data.

**Dataset S1 (separate file).**  Study 2 data.

All data, materials, and pre-registrations are available on the Open Science Framework (OSF):<https://osf.io/f6gmb/?view_only=2ad7305cce094ff4a349850dcbcc304e>.
